# Impact of Post-Traumatic Stress Disorder Management Through Reconsolidation Therapy on Fibromyalgia Syndrome: A Pilot Study

**DOI:** 10.3390/biomedicines14010190

**Published:** 2026-01-15

**Authors:** Ghina Harika Germaneau, Delphine Rannou, Elodie Charrier, Yassir El Fairouqi, Alain Brunet, Damien Doolub, Nicolas Langbour, Isabelle Raviart, Issa Wassouf, Nemat Jaafari

**Affiliations:** 1 Centre de Recherches sur la Cognition et l’Apprentissage, CNRS UMR 7295, Université de Poitiers, Université de Tours, 86000 Poitiers, France; 2 Unité de Recherche Clinique Pierre Deniker, Centre Hospitalier Henri Laborit, 86000 Poitiers, France; 3 Centre Régional d’Étude et de Traitement de la Douleur, CHU de Poitiers, 86000 Poitiers, France; 4 Centre Hospitalier du Nord Deux-Sèvres, Service de Psychiatrie Adulte, 79100 Thouars, France; 5 The Australian National PTSD Centre, The University of the Sunshine Coast Thompson Institute, Birtinya, QLD 4575, Australia; 6 McGill’s Psychiatry Department, Douglas Institute Research Center, 6875 Lasalle Boulevard, Montreal, QC H4H 1R3, Canada

**Keywords:** PTSD, fibromyalgia, reconsolidation therapy, stress-related neurobiology, quality of life, depression

## Abstract

**Background**: Fibromyalgia syndrome (FMS) and post-traumatic stress disorder (PTSD) may co-occur and are associated with increased symptom burden, functional impairment, and reduced quality of life. Accumulating evidence suggests shared neurobiological mechanisms. Trauma-focused interventions targeting maladaptive memory processes may therefore represent a relevant therapeutic approach in this population. **Objective**: To evaluate the feasibility, tolerability, and preliminary clinical associations of a brief reconsolidation-based therapy in women with comorbid FMS and PTSD. **Methods**: This multicenter pilot study included adult women diagnosed with FMS and PTSD who underwent six sessions of reconsolidation therapy combining traumatic memory reactivation with propranolol administration. Clinical outcomes were assessed at baseline and at 3-month follow-up using the Fibromyalgia Impact Questionnaire (FIQ), the Impact of Event Scale–Revised (IES-R), the Montgomery–Åsberg Depression Rating Scale (MADRS), the Beck Depression Inventory (BDI), the Rosenberg Self-Esteem Scale (RSES), and the SF-36. Changes over time were analyzed using paired statistical tests and linear mixed-effects models. **Results**: Fourteen participants completed the intervention and follow-up assessments. The intervention was feasible and well tolerated. Changes over time were observed in fibromyalgia-related quality of life (FIQ scores), PTSD symptom severity (IES-R), and depressive symptoms (MADRS, BDI), as well as in selected SF-36 domains, including vitality, social functioning, and mental health. A progressive decrease in IES-R scores was observed across treatment sessions. **Conclusions**: This pilot study suggests that reconsolidation-based therapy is feasible in women with comorbid FMS and PTSD and was associated with changes in PTSD symptoms and fibromyalgia-related functional impact. Given the exploratory design and absence of a control group, these findings should be interpreted cautiously and warrant confirmation in larger, controlled trials.

## 1. Introduction

Fibromyalgia (FM) is a chronic pain syndrome characterized by widespread musculoskeletal pain, fatigue, sleep disturbances, and cognitive dysfunction. Its heterogeneous presentation significantly impairs daily functioning and social participation, leading to reduced quality of life. FM affects 2–4% of the population [1], with a predominance in women. In France, prevalence is estimated at 1.6% of adults, or 1.2 million individuals [2].

Although its pathophysiology remains unclear, FM is strongly associated with central sensitization, whereby altered pain processing within the central nervous system amplifies sensory perception. Dysfunction of descending pain modulatory pathways, involving impaired inhibitory control and altered brainstem–cortical interactions, has been identified as a key neurobiological feature of FM [3]. A biopsychosocial model describes predisposing, triggering, and perpetuating factors contributing to symptom persistence [4,5]. Among psychosocial contributors, trauma and major stressors are frequent reported and patients with FM report higher rates of trauma exposure, including abuse, compared with controls [6,7]. The association between trauma exposure and FM appears largely mediated by post-traumatic stress disorder (PTSD) [8,9].

From a neurobiological perspective, both FM and PTSD have been associated with dysregulation of stress-response systems, including alterations of the hypothalamic–pituitary–adrenal axis, autonomic nervous system imbalance, and neuroinflammatory processes. These abnormalities are thought to contribute to persistent hyperarousal and maladaptive pain modulation through sensitization of cortico-limbic and brainstem networks involved in threat detection, emotional memory, and nociceptive processing. Recent evidence highlights the contribution of psychological factors such as anxiety and pain acceptance in modulating the impact of trauma on pain perception and disability in fibromyalgia and related conditions [10]. Trauma-related alterations in emotional memory circuits may therefore represent a key biological link between PTSD and FM, providing a mechanistic framework for investigating trauma-focused interventions in this population.

PTSD is a psychiatric disorder marked by intrusive memories, avoidance, hyperarousal, and emotional dysregulation. It involves stress-related brain circuits, particularly the hypothalamic–pituitary–adrenal (HPA) axis and limbic structures, which also contribute to pain regulation [11].

PTSD prevalence in FM is 39.1%, with lifetime rates up to 56% [12,13,14,15]. This co-occurrence aggravates symptoms, reduces quality of life, and worsens treatment outcomes [16,17]. Shared mechanisms include HPA axis dysregulation, autonomic dysfunction, neuroinflammation, and maladaptive plasticity. Early-life adversity, chronic stress, and catastrophizing further intensify symptoms.

Despite these associations, trauma-focused treatments are not included in FM guidelines. Cognitive-behavioral therapy (CBT) is moderately recommended in FM [18] and first-line for PTSD, yet shows limited benefit in comorbid patients. FM-related fatigue, catastrophizing, and somatic preoccupation hinder engagement in cognitive restructuring and exposure [19], and adherence is often poor.

Alternative trauma-focused approaches adapted to FM/PTSD are therefore needed. Reconsolidation therapy [20,21,22] targets traumatic memories by disrupting their reconsolidation. The protocol combines memory reactivation with propranolol, a beta-blocker that blunts the emotional and physiological stress response. This reduces the distress associated with the memory, making it more tolerable.

Evidence suggests reconsolidation therapy yields rapid improvements in PTSD symptoms, is well tolerated due to lower emotional intensity, and has reduced dropout rates. The protocol is simple, requiring a single dose of propranolol with memory reactivation, and can be implemented in outpatient and pain management settings with minimal resources.

The present pilot study aims to evaluate whether reconsolidation blockade therapy improves fibromyalgia-related quality of life, as measured by the FIQ.

## 2. Materials and Methods

### 2.1. Study Design

We conducted this multicentric pilot study in two hospitals (Poitiers and Thouars). Data were collected from August 2021 to January 2025.

### 2.2. Ethics Approval

The study design was approved by the French Regional Ethics Committee (11 May 2021, n° 21 04 09) and Drug Regulatory Agency (19 May 2021). Registration of this study was made on the clinical trial registry before the start of the study (ClinicalTrial.gov, identifier: NCT04950426). All experiments were performed following the relevant guidelines and regulations. All participants were naïve to the purpose of the study and written informed consent was obtained prior to inclusion.

### 2.3. Participants

Two senior psychiatrists recruited participants diagnosed according to DSM-5 criteria. Eligible participants were adults aged 18 years or older with a diagnosis of fibromyalgia syndrome based on the 2016 ACR criteria, and a comorbid diagnosis of PTSD, defined by meeting DSM-5 criteria following exposure to a single or repeated traumatic event. Participants were required to have a PCL-5 (PTSD Checklist for DSM-5) score greater than 33, indicating clinically significant PTSD symptoms. Patients were included only if they had stable pain management and/or psychotropic treatments for at least two months, were able to read and understand French.

Patients were excluded if they presented a severe psychiatric or neurological disorder (including bipolar or psychotic disorders, acute suicidal, homicidal, or self-injuring intent, traumatic brain injury within the last five years, or alcohol or substance dependence), were involved in trauma-related legal proceedings, or were pregnant or breastfeeding. Additional exclusion criteria included unstable psychotropic treatment, any contraindicated drug interaction with propranolol, a resting heart rate below 55 beats per minute, a systolic blood pressure below 100 mmHg, or any other medical condition considered contraindicating by the study physician.

### 2.4. Procedure

After receiving detailed study information, participants provided written informed consent. Eligibility was then confirmed by a clinician prior to inclusion. Treatment was initiated within 7 days of enrollment. Six weekly Reconsolidation Therapy™ (RT) sessions were scheduled, with an intersession interval ranging from 4 to 15 days.

At baseline, participants underwent a medical assessment including a pregnancy test, electrocardiogram (ECG), MINI-S interview (Mini International Neuropsychiatric Interview—Suicidality module) [23], and the Posttraumatic Stress Disorder Checklist for DSM-5 (PCL-5) [24]. Clinician-rated measures (Fibromyalgia Impact Questionnaire [FIQ] [25], Montgomery–Åsberg Depression Rating Scale [MADRS] [26], Clinical Global Impressions–Severity [CGI-S]) [27] and self-reported measures (Beck Depression Inventory [BDI] [28], Rosenberg Self-Esteem Scale [RSES] [29], and Short Form-36 Health Survey [SF-36]) [30] were administered. Pain distribution was assessed using a standardized body map. All assessments were repeated at the 3-month endpoint.

### 2.5. Study Treatment

Reconsolidation Therapy™ consists of the structured reactivation of the patient’s most distressing traumatic memory, combined with the administration of the β-adrenergic blocker propranolol. The intervention comprises six weekly sessions, each lasting approximately 10 to 25 min and delivered by a trained therapist. During the first session, participants compose a 1–2 page written narrative of their traumatic experience. From sessions 1 to 6, patients read this narrative aloud. In subsequent sessions, participants may revise or expand the narrative to include additional distressing details that emerged during the preceding week.

The narrative is required to provide a detailed account of the traumatic event, with particular emphasis on its most emotionally intense component (“hot spot”), to describe at least five associated somatic sensations, and to be written in the first-person, present tense [20].

Before each session, blood pressure and heart rate are measured. An oral dose of propranolol (1 mg/kg, calculated according to ideal body weight) is administered 75 min (±15 min) prior to memory reactivation. Following each session, concomitant treatments are documented, adverse events are monitored, suicide risk is assessed, and trauma-related symptom severity is evaluated using the Impact of Event Scale–Revised (IES-R). The IES-R is a 22-item self-report questionnaire assessing post-traumatic symptom severity on a 5-point Likert scale ranging from 0 (“Not at all”) to 4 (“Extremely”).

### 2.6. Outcomes Assessment

All clinical outcomes were assessed at baseline (T0) and at the predefined study endpoint, corresponding to the 3-month follow-up visit (T3). The primary outcome was the change in quality of life in patients with fibromyalgia syndrome, measured by the FIQ between baseline (T0) and the 3-month follow-up (T3). Secondary outcomes included changes between baseline (T0) and the 3-month follow-up (T3) in PCL-5, MADRS, BDI, SF-36, RSES, and CGI scores. Changes in IES-R scores were analyzed longitudinally across the six treatment sessions. Pain maps were generated at baseline (T0) and at the 3-month follow-up (T3). For each anatomical region, the number of patients reporting pain was recorded, and composite maps were created to represent the spatial distribution of pain across the study population.

### 2.7. Statistical Analyses

All statistical analyses were performed using the Jamovi Project (2024), Jamovi (Version 2.5), https://www.jamovi.org (Accessed on 2 May 2025).

Continuous variables were summarized as mean ± standard deviation. The level of statistical significance was set at *p* < 0.05 (two-tailed).

Normality of data distribution was assessed using the Shapiro–Wilk test and visual inspection of Q–Q plots. Clinical outcome measures were compared between baseline (T0) and 3-month follow-up visit (T3) using paired Student’s *t*-tests when normality assumptions were met or Wilcoxon signed-rank tests otherwise.

The evolution of IES-R scores across treatment sessions was analyzed using a linear mixed-effects model, with session treated as a fixed effect and subject as a random intercept. Post hoc comparisons were performed using Tukey’s multiple-comparison procedure.

## 3. Results

**Figure 1:**
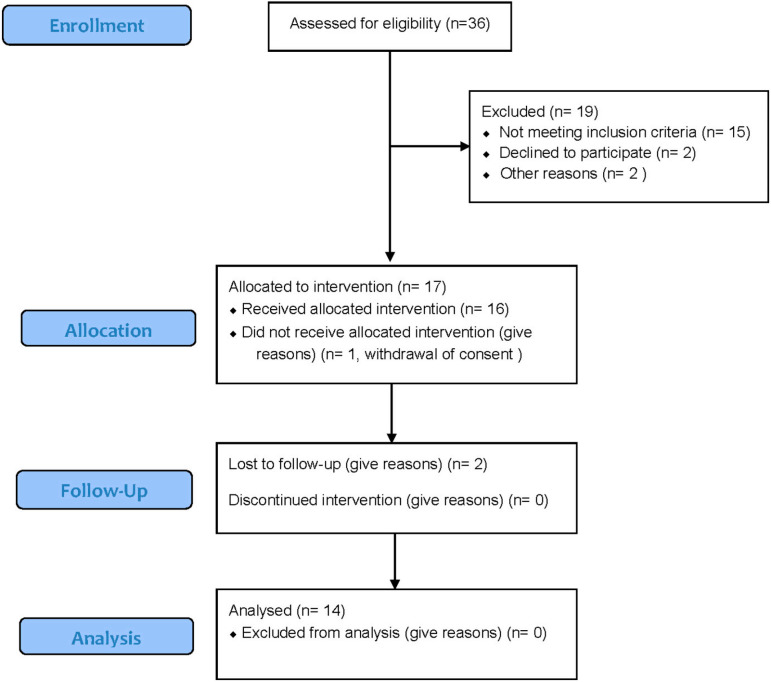
Flow diagram.

In this pilot study, 17 women were enrolled. One participant did not receive the intervention and two were lost to follow-up (Figure 1). Analyses were therefore conducted on 14 completers. Participants were aged between 24 and 62 years (mean age = 49.2 ± 9.9 years) and had been diagnosed with fibromyalgia for a mean duration of 6.0 ± 6.4 years (range: 1–24 years). Seven participants were married or living with a partner, and 11 had completed at least a bachelor’s degree.

Regarding trauma history, four participants reported childhood or adolescent sexual assault, one reported adult rape, one reported childhood physical violence from a parent, one reported the traumatic suicide of her father, two reported violence from a former partner, two reported moral and sexual harassment by an employer, one reported a complicated abortion, one reported perinatal loss, and one had experienced a road traffic accident (Table 1).

**Figure 2:**
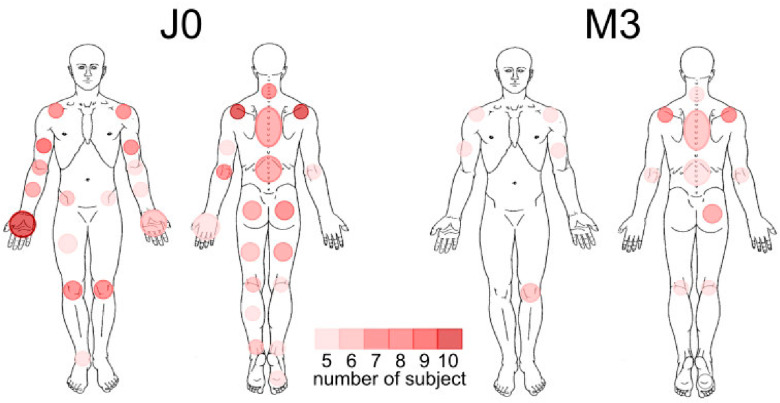
Pain distribution maps at baseline (T0) and 3-month follow-up (T3).

After the intervention, changes over time were observed in fibromyalgia-related quality of life as measured by the FIQ, as well as reductions in PTSD symptom severity (Table 2). These changes were also illustrated by the comparison of pain mapping at baseline (T0) and at 3-month follow-up (T3) (Figure 2). Given the exploratory nature of the study, these findings should be interpreted cautiously.

Changes were also observed in depressive symptoms, as measured by both the MADRS and the BDI, along with an increase in self-esteem (Table 2).

Regarding health-related quality of life changes over time were particularly observed in the SF-36 domains of General Health, Vitality, Social Functioning, Role–Emotional, and in the Mental Component Summary score (Table 2).

**Figure 3:**
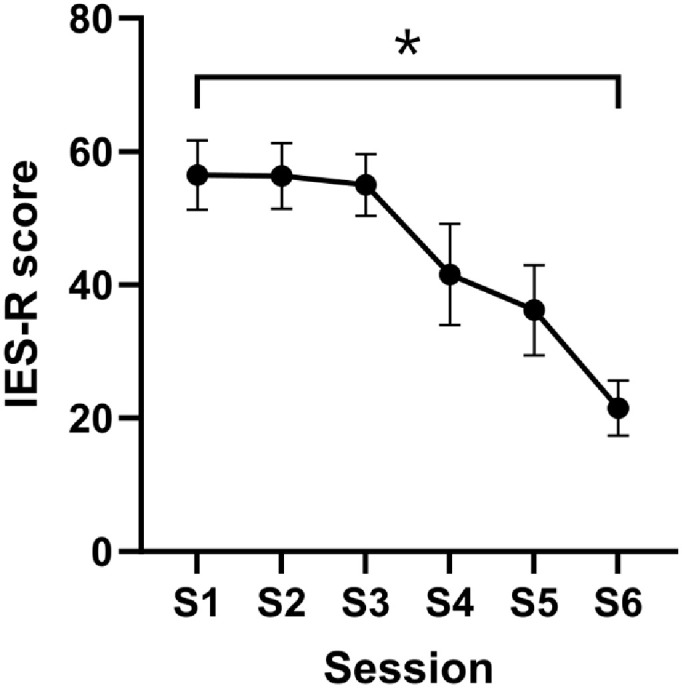
Trajectories of IES-R scores across treatment sessions. Values represent mean ± SEM. * indicates a statistically significant decrease between session 1 and session 6 (*p* < 0.05).

A significant decrease in IES-R scores was observed between session 1 and session 6 (corrected *p* = 0.0476), reflecting a reduction in subjective post-traumatic stress related to traumatic memories (Figure 3). Given the small sample size, and the absence of a control group all analyses should be considered exploratory.

Regarding pharmacological treatments, non-opioid analgesics were the most frequently prescribed medications (56.2%). Benzodiazepines were used by 44% of participants, indicating frequent use of anxiolytic treatments. Antidepressants (SSRIs, SNRIs, and tricyclic) were prescribed in the majority of cases (11/16 patients, 68.7%). The use of opioids and hypnotic medications suggested concurrent management of chronic pain and sleep disturbances.

## 4. Discussion

The present pilot study provides preliminary evidence supporting the feasibility and potential clinical relevance of a short, trauma-focused reconsolidation-based therapy in women with comorbid fibromyalgia syndrome (FMS) and post-traumatic stress disorder (PTSD). Following a brief intervention, participants showed changes in PTSD symptom severity, fibromyalgia-related functional impact, depressive symptoms, and several domains of quality of life.

The co-occurrence of PTSD and fibromyalgia has been increasingly recognized, with prevalence estimates ranging from 20% to 40% in fibromyalgia populations [8,9]. This association is thought to reflect shared pathophysiological mechanisms, including hypothalamic–pituitary–adrenal (HPA) axis dysregulation, central sensitization, altered pain modulation, and autonomic nervous system hyperreactivity [31]. In particular, dysfunction of descending pain modulatory systems and interactions between stress-related emotional memory circuits and central sensitization processes may provide a biological framework for understanding the observed changes in fibromyalgia-related functional impact following trauma-focused interventions [3]. These overlapping neurobiological processes provide a plausible framework for understanding why trauma-focused interventions may have downstream effects on pain processing, fatigue, sleep disturbances, and overall disability [10].

The observed reduction in FIQ scores in the present study suggests a potentially clinically meaningful change in functional status, although these findings should be interpreted cautiously given the pilot design. The average change exceeded commonly accepted thresholds for minimal clinically important differences [32], although participants generally remained within the moderate-to-severe impact range.

Improvements in fibromyalgia-related impact were accompanied by gains in health-related quality of life, as reflected by changes in SF-36 domains, particularly vitality, social functioning, emotional role, and mental health. While fibromyalgia-specific instruments are often correlated with generic quality-of-life measures, it is important to distinguish between the original FIQ developed by Burckhardt et al. [25] and the revised FIQR published in 2009 by Bennett et al. [32]. The FIQR, rather than the original FIQ, has been more extensively validated against multidimensional instruments such as the SF-36 due to its domain-based structure and improved psychometric performance [33]. In this study, the use of the original FIQ limits the precision of cross-instrument comparisons.

Beyond improvements in PTSD symptoms, participants exhibited reductions in depressive symptom severity and enhancements in self-esteem, suggesting broader effects on affect regulation and self-perception. These findings are consistent with the conceptualization of PTSD as a disorder of maladaptive threat processing [34] and emotional memory [35].

Previous studies have demonstrated that cognitive-behavioral therapy (CBT) can be beneficial in fibromyalgia and in patients with comorbid trauma histories [36,37]. However, CBT mainly targets cognitive appraisals and coping strategies through top–down mechanisms [38,39], whereas reconsolidation-based interventions are hypothesized to act through bottom–up neurobiological processes involving memory reactivation and interference with memory restabilization [40,41,42]. Clinical work using propranolol-supported reconsolidation has shown promising results in PTSD populations [20,21].

This theoretical distinction may be particularly relevant in fibromyalgia, a condition characterized by heightened central nervous system reactivity and stress-related hyperarousal [43,44]. By attenuating limbic and autonomic activation, reconsolidation-based approaches may indirectly reduce hypervigilance, muscular tension, and pain catastrophizing, thereby contributing to improvements in pain-related disability [45,46]. Nevertheless, direct comparative trials between reconsolidation therapy and CBT are currently lacking.

Several clinical models have been proposed to address the co-occurrence of PTSD and chronic pain, including sequential, parallel, and integrated treatment approaches [47]. Existing studies have evaluated emotional exposure-based therapies, integrated CBT protocols, multidisciplinary care models, and eye movement desensitization and reprocessing (EMDR) [36,48,49]. Systematic reviews indicate that trauma-focused psychological interventions reliably reduce PTSD symptoms, while their effects on pain outcomes are more variable [50,51].

This study has important limitations. The small sample size, lack of a control group, and exploratory design limit causal inference and generalizability. The absence of blinded outcome assessment increases the potential for expectancy effects. Heterogeneity of trauma histories and concomitant pharmacological treatments may also have influenced outcomes. In addition, the timing of assessments should be more clearly standardized.

Furthermore, the study did not include objective or semi-objective measures of pain intensity, sleep quality, or fatigue severity, which are central dimensions of fibromyalgia and should be systematically assessed in future studies [8,52,53].

Despite these limitations, the present findings support the feasibility and acceptability of reconsolidation-based therapy in women with comorbid FMS and PTSD. Larger, adequately powered randomized controlled trials are now required to confirm efficacy, clarify mechanisms of action, identify predictors of response, and determine how trauma-focused interventions can be optimally integrated into multidisciplinary fibromyalgia care pathways.

## 5. Conclusions

This pilot study provides preliminary evidence supporting the feasibility and potential clinical value of reconsolidation therapy as a trauma-focused intervention for women with comorbid fibromyalgia and PTSD. Given the chronic and complex nature of both conditions, this approach may offer a brief, well-tolerated, and accessible therapeutic option that could be integrated into pain and mental health care settings.

Nevertheless, the absence of a control group, the small sample size, and the exploratory nature of this study limit the strength of causal inferences and generalizability. Larger, adequately powered randomized controlled trials are now required to confirm efficacy, clarify mechanisms of action, assess durability of effects, and determine how trauma-focused interventions can be optimally incorporated into multidisciplinary fibromyalgia care pathways. These findings do not allow conclusions regarding clinical efficacy.

## Figures and Tables

**Table 1:** Types of trauma.

Patient	Types of Trauma	Patient	Types of Trauma
01	Father’s suicide	10	Adolescent rape
02	Intrafamilial sexual abuse (sibling)	11	Road traffic accident
03	Spouse’s stroke	12	Complicated abortion
04	Intrafamilial abuse involving a child	13	Workplace sexual harassment
05	Assault by employer	14	Adult rape
06	Death threats from ex-partner	15	Death of one’s baby at birth
07	Domestic violence	16	Workplace harassment
08	Violence from father	17	Childhood rape
09	Childhood rape by an uncle		

**Table 2:** Clinical outcomes.

	Baseline (T0)	3 Month Follow-Up (T3)	*p*-Value
FIQ score	65.2 ± 12.8	53.5 ± 18.3	0.018
PCL-5 score	50.1 ± 9.5	25.9 ± 14.2	<0.001
RSES score	21.2 ± 5.3	24.6 ± 5.6	0.032
MADRS score	15.5 ± 6.6	8.6 ± 4.3	<0.001
BDI score	17 ± 3.5	12 ± 6.1	0.003
CGI-S score	4.8 ± 0.5	3.9 ± 0.9	0.002
CGI-I		2.0 ± 0.9	
SF-36			
Physical Functioning	47.2 ± 14.6	53.9 ± 19.2	0.088
Role-Physical	15.6 ± 27.2	25 ± 31	0.466
Bodily Pain	29.1 ± 14.8	34.3 ± 14.9	0.146
General Health	36 ± 15.4	42.7 ± 20.4	0.047
Physical Health Component	23.8 ± 1	24.2 ± 1.2	0.169
Vitality	17.2 ± 11.8	30.7 ± 24.9	0.023
Social Functioning	37.5 ± 20.4	55.4 ± 20.6	0.008
Role-Emotional	14.6 ± 29.7	47.6 ± 42.8	0.026
Mental Health	59.5 ± 9.5	63.4 ± 4.4	0.183
Mental Health Component	22.2 ± 0.9	22.9 ± 0.7	0.029

Notes: Data are presented as mean ± SD. FIQ: Fibromyalgia Impact Questionnaire; PCL-5: Posttraumatic Stress Disorder Checklist for DSM-5; RSES: Rosenberg Self-Esteem Scale; MADRS: Montgomery-Asberg Depression Rating Scale; BDI: Beck Depression Inventory; CGI-S: Clinical Global Impressions Severity; CGI-I: Clinical Global Impressions Improvement; SF-36: Short-Form 36 Health Survey. *p*-values were obtained using paired *t*-tests or Wilcoxon signed-rank tests, as appropriate.

## Data Availability

The data presented in this study are available on reasonable request from the corresponding author.
